# The Implementation of a Low Power Environmental Monitoring and Soil Moisture Measurement System Based on UHF RFID

**DOI:** 10.3390/s19245527

**Published:** 2019-12-14

**Authors:** Žiga Korošak, Nejc Suhadolnik, Anton Pleteršek

**Affiliations:** 1 STMicroelectronics d.o.o., Tehnološki park 21, 1000 Ljubljana, Slovenia; ziga.korosak@st.com (Ž.K.); nejc.suhadolnik@st.com (N.S.); 2 Faculty of Electical Engineering, Tržaška cesta 25, 1000 Ljubljana, Slovenia

**Keywords:** RFID, moisture sensor, low power microcontroller, sensors, data acquisition, precision agriculture, relative humidity, UHF tag, environmental monitoring

## Abstract

A smart sensor label based on the integration of ultra high frequency (UHF) radio frequency identification (RFID) technology and sensors is presented. The label is composed of a semi-active system that measures temperature, light, relative humidity and gravimetric water content (GWC) in the soil. The deployed system provides a simple, cost effective solution to monitor and control the growing of plants in modern agriculture and is intended be a part of a smart wireless sensor network (WSN) for agricultural monitoring. This paper is focused on analysis and development of a moisture sensor to measure GWC. It is based on a capacitance measurement solution, the accuracy of which is enhanced using several sensor driving frequencies. Thanks to the cancellation of supply voltage variations, the modeling of the GWC sensor and readout circuit was correct. The results we measured were close to modeled values. The maximum measurement resolution of the capacitive moisture sensor was 0.07 pF. To get the GWC from measured capacitance, a scale was used to weigh the mass of water in the soil. The comparison between capacitance measurement and calculated soil GWC is presented. The RFID measurement system has energy harvesting capabilities and an ultra-low power microcontroller, which uses embedded software to control the measurement properties. The microcontroller has to choose the appropriate model depending on the measured amplitude and chosen frequency to calculate the actual voltage on the sensing capacitor.

## 1. Introduction

Radio-frequency identification (RFID) devices come in two types: (1) readers that generate magnetic fields or electromagnetic (EM) waves from their antennas and issue commands, and (2) tags that are powered from the reader’s magnetic field or EM waves and respond to its commands. Tags can be passive or active. Passive tags are powered entirely by the reader and respond to the reader by modulating the load on their antenna, whereas active tags have their own power source and can, along with modulating antenna load, also modulate the field above the reader’s level by using their own power. The frequency range of RFID is very wide, with the majority of devices falling into one of the following bands: low frequency (LF), high frequency (HF) or ultra high frequency (UHF). LF ranges from 30 kHz to 300 kHz, with the most common operating frequency at 125 kHz. HF ranges from 3 MHz to 30 MHz, with the most common operating frequency at 13.56 MHz. And finally, UHF ranges from 300 MHz to 3 GHz, with the most common operating frequency range from 860 MHz to 960 MHz.

Many RFID tags also offer energy harvesting capabilities, as the power of the radio-frequency (RF) field can be higher than what the tag needs for its operation. External sensors or microcontrollers can be powered by the tag’s harvested energy. Additionally, tags can have wired interfaces to communicate with these external devices. Devices with the extended capabilities of supporting external sensors, communicating with microcontrollers or including internal sensors are often called smart tags or smart active labels (SAL).

The field of smart agriculture started developing back in 1980s with sensing parameters of the soil [1]. The field has quickly diversified into many technologies, such as satellite imaging, light detection and ranging, fluorescence spectroscopy and RFID [2]. Since then several papers have been published demonstrating the usefulness of smart RFID tags in agriculture. Advances in RFID technology have allowed vast opportunities for research, development and innovation in the field. An overview of the current state of the field can be found in [2]. RFID technology has also been combined with wireless sensor networks (WSNs) in systems based on web services to allow for traceability of collected data [3]. Such systems could be combined with the proposed solution to achieve traceability of collected data to its precise geographical location. The use of unmanned aerial vehicles (UAV) has been explored in conjunction with RFID and WSN technologies to achieve a remote or automatic operation of the sensor network with limited communication range [4]. This idea is very applicable in this work, as the sensors could be read out with a reader attached to a UAV. Once landed the attached reader could transfer sensor data to a web service, that would create a map of measured parameters, and calculate when to water crops, apply pesticides, etc. Others have also demonstrated the use of soil moisture sensors with near-field communication (NFC) devices [5] and UHF RFID devices [6]. RFID technology can also be used for other sensor applications, as has been demonstrated by [7]. None of above mentioned sensor systems, however, are able to operate independently due to their passive nature. Additionally, monitoring soil parameters in the field with an NFC device is impractical due to their short range (up to 10 cm). This range is further reduced when powering external sensors in addition to the tag itself, and can go as low as 1 cm, as demonstrated in [7].

In this paper we provide an example of a semi active UHF RFID based sensor system for monitoring soil moisture and environmental parameters including temperature, humidity and light. The measurement data is stored in the electrically erasable read only memory (EEPROM) of the smart tag. The data can be used not only to plan irrigation, but also to estimate the growth cycle of the crop, and plan fertilizing, and even harvesting. Tags installed over a large area of land could easily be read by a UAV attached reader flying overhead due to UHF RFID’s superior range over NFC. Commercial UHF RFID readers can be found with read ranges of up to 12 m [8] or up to 7 m [9] in handheld format.

The paper is organized as follows. Section 2 provides an overview and description of the system with a focus on its low power capabilities. Section 3 takes a detailed look at the soil moisture measurement solution based on [5] and improves upon it. In Section 4 results of measurements are presented. Lastly, conclusions are presented in Section 5.

## 2. The Measurement System

In this section the developed system will be described and its power consumption measured.

### 2.1. System Description

**Figure 1:**
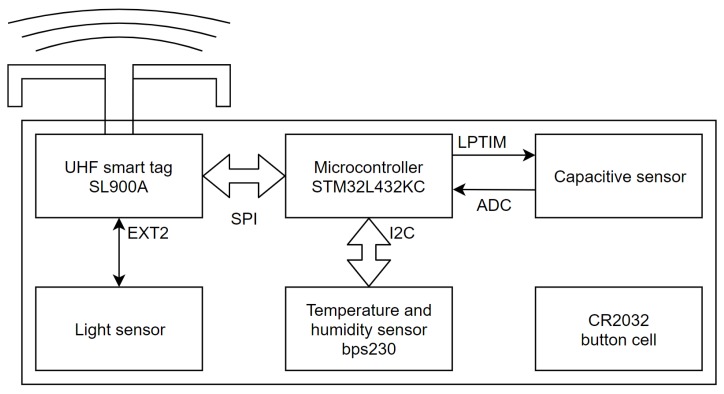
System block diagram.

The system designed in this work is shown in Figure 1.

**Figure 2:**
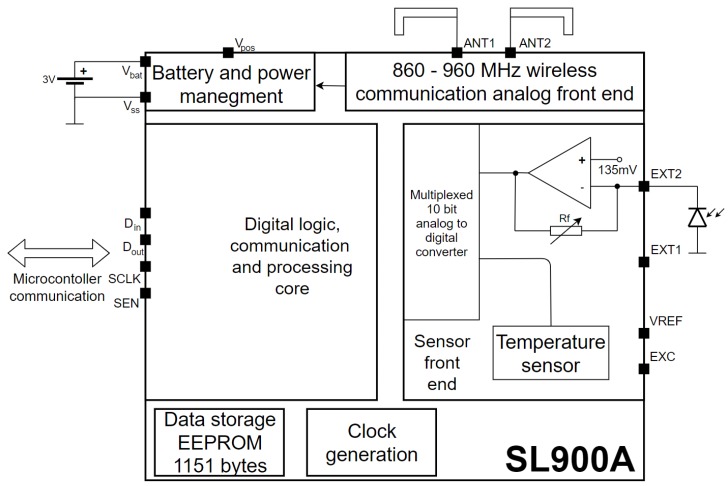
Block diagram of SL900A with immediate surroundings.

It is composed of an SL900A [10] smart tag used for communicating with an RFID reader, logging temperature and light data and storing it in its EEPROM memory. The device features a serial peripheral interface (SPI), which is used to communicate with the microcontroller. It is compatible with EPC Gen2 [11] command protocol and features additional cool-Log™commands enabling the chip to be used for automated sensing on its own. The internal block diagram of the SL900A with its immediate surroundings is shown in Figure 2.

The SL900A also supports energy harvesting, but it was not used for that in our study, due to a severe reduction in range when powering the system via RF EM waves and an inability to operate without an RFID reader present. SL900A was used along with its evaluation board, which provides an antenna and a slot for LR44 button cell, which was not used. For the microcontroller, STM32L432KC [12] was chosen because of its ultra-low power capabilities. It was used along with its Nucleo L432KC development board. It can operate with clocks up to 80 MHz and uses just 280 nA in standby mode with real time clock and 94 μA/MHz in low power run mode. It features multiple digital interfaces, including a SPI and inter-integrated circuit $\left(I^{2}C\right)$; an internal RC oscillator that can be calibrated with a real time clock external crystal oscillator; a 12 bit successive approximation analog-to-digital converter (ADC); and multiple timers, including two low power 16 bit timers (LPTIM). The BPS230 [13] digital temperature and humidity sensor was chosen for our humidity sensor. It has a wide supply voltage range operating from 1.62 to 5.5 V and typically uses just 10 nA in sleep mode and 13 μA in operating mode. The humidity measurement is automatically corrected with the temperature measurement and has 0.1% relative humidity resolution with 5% accuracy on the whole relative humidity range at temperatures from 5 °C to 45 °C, and 3% accuracy from 20% to 80% relative humidity at 25 °C. For the light sensor, VEMD6060x01 [14] from Vishay Semiconductors was used. To communicate with the measurement system an IDS-DK-R902-LP2 UHF RFID reader evaluation kit supporting EPG gen2 was used, along with python scripts for initializing the measurement system and reading the measurement data. The procedure to read data is described in the next subsection.

### 2.2. System Power Consumption

**Figure 3:**
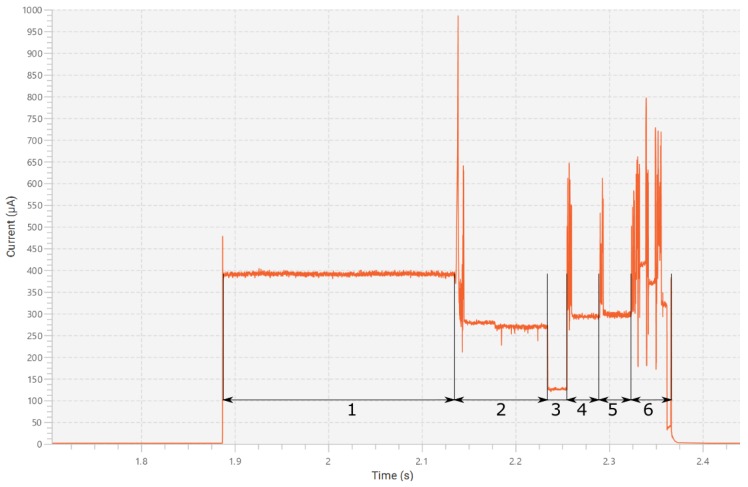
Power consumption during the measurement operation.

Total system power usage was measured using X-NUCLEO_LPM01A STM32 Power shield [15]. It is able to measure currents in the range from 100 nA to 50 mA with 2% accuracy and sampling rates from 1 Hz to 100 kHz. The device also has an optional external trigger that can be triggered by the device being tested. The power consumption graph during the measurement operation is displayed in Figure 3. In order to measure the power usage of the entire system and not just the Nucleo board, the entire system was connected to the power shield using power and ground wires. The system was then activated to log the power used during two modes of operation: during the measurement operation and during transaction with an RFID reader. In Figure 3 current consumption during the measurement operation is shown.

In the first part of the Figure, the system is idle in shutdown mode. Power consumption in this mode around is 1 μA. When a wake up signal is received at 1.89 s the system first turns on an indicator light emitting diode for 250 ms. This part is marked with number 1 on the graph and is for user signaling and debugging. As this is not strictly necessary it can be easily removed when it is not needed. At 2.14 s the actual measurement operation commences (numbers 2–5 on the graph). First, BPS230 is enabled and command is issued for it to start measuring. This measurement operation is marked with number 2 on the graph. At 2.24 s BPS230 finishes its measurement, which causes a significant drop in power. The microcontroller is programmed with a fixed delay here, so it sleeps until 2.25 s on the graph. This non active part is marked with number 3 on the graph. At this point the data from BPS230 is gathered and processed. BPS230 is disabled and the capacitive moisture sensor is powered. Note that powering on the capacitive sensor and BPS230 simultaneously was avoided due to noise issues. Initially the drive frequency was 250 kHz. The sensor was given 30 ms to stabilize. This wait is marked with number 4 on the graph. At 2.29 s the stabilization is finished and data is read out with ADC. At this point data is checked to make sure it is in an appropriate range. When that is not the case, another drive frequency is chosen depending on the data and the measurement is repeated. The second stabilization phase is marked with number 5 on the graph. At 2.32 s both measurements are finished, and communication is initiated with SL900A. Temperature sensor data, light sensor data and EEPROM contents are read. All sensor data is then packaged along with time data into 9 bytes and written into memory. A wake up alarm is set and system is shut down. This whole last section is marked with number 6 on the graph. The spikes at various events happen due to processing or digital communication. Note that during various operations the microcontroller is in the lowest power state permitted during those operations. The entire measurement operation lasts 0.25 s and consumes 227 μJ of energy at 3.3 V supply voltage. The signaling operation adds 0.25 s of time and consumes 320 μJ of energy. During combined cycle of both operations the average current is 334 μA, accounting for 167 μAs of charge used. Even with combined power usage of those two, the system can perform over 3 million measurements with one standard CR2032 button cell [16] or stay idle for over 20 years assuming the battery does not fail due to age.

**Figure 4:**
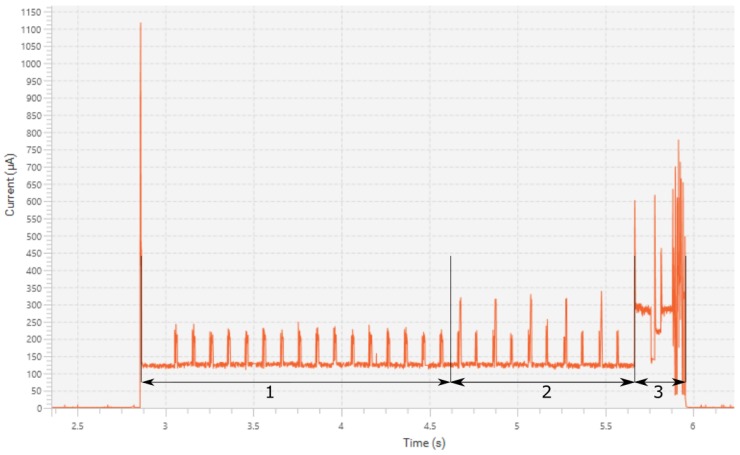
Power consumption during the measurement operation.

Current consumption during transaction with the RFID reader is shown in Figure 4. The first current spike in the figure happens when the reader turns on its EM field. At this point $V_{\mathrm{pos}}$ pin on SL900A goes high which wakes up the microcontroller from shutdown. The microcontroller then checks what the source of this wake up was, sees it was from $V_{\mathrm{pos}}$ interrupt and then checks if $V_{\mathrm{pos}}$ is still high. If that is still the case the microcontroller sets an interrupt on the falling edge of $V_{\mathrm{pos}}$ and goes to stop mode. During the time when the microcontroller is in stop mode the transaction happens. The transaction is initiated by the reader performing inventory round. After that, a standard EPC Gen2 [11] read command is issued. The read command reads 32 blocks of EEPROM totaling 64 bytes. Packets of communication when EEPROM is read can be clearly seen on the graph, as marked with number 1. There are 16 packets of read data in total. After EEPROM is read, a new configuration is written to the first 10 bytes of EEPROM. This is done by issuing EPC Gen2 write commands. Each command writes one block or 2 bytes. Five write commands are issued in total, with each followed by a read command to check what was written. Write commands can be seen as higher spikes on the graph with the lower spikes being read commands. This section is also marked with number 2 on the graph. At the last current spike the reader field is turned off. At this point the microcontroller wakes up, reads configuration bytes from SL900A and sees if there was a configuration change. If the configuration is changed, a measurement is performed after the readout. This can be seen on the graph as marked with number 3, with this measurement skipping the signaling phase. The entire readout operation lasts 1.8 seconds, and consumes 804 μJ at 3.3 V supply voltage. The average current during the readout operation is 135 μA. The write operation lasts 1.6 seconds and consumes 753 μJ at 3.3 V supply voltage. The average current during the write operation is 136 μA. Both operations combined consume about the same amount of energy as three measurement operations.

As SL900A can operate passively, measurement data can be read with a RFID reader even if the battery has been drained. It responds to read command with its EEPROM contents whether battery power is present or not. When the reader finishes gathering data from SL900A it sends it to a personal computer where it is unpacked as follows. This step remains the same regardless whether a hand held reader or a UAV mounted reader that reads many such systems before returning with the data is used. The first 10 bytes are configuration bytes. After that, each 9 bytes represent a measurement result. In those 9 bytes, the first 24 bits are the time stamp of the measurement. That is in relative time format, measured from the configured start time to save space. The next 10 bits are for relative humidity. This is saved in fixed point format, as 10 bits give 0.1% of resolution. Temperature follows with 12 bits. Then, light gets an additional 12 bits; and lastly, moisture sensor data are written with 2 bits for range data and 12 bits for result. The result is saved in the form of measured amplitude in percent offset by 60 to get range 0–40%. With 12 bits this gives 0.01% resolution, which is more than required but was used in order to get full 9 bytes of result data. After moisture data is decoded it is converted into the actual capacitance value using the model described in Section 3.1.

## 3. Capacitive Soil Moisture Measurement Solution

**Figure 5:**
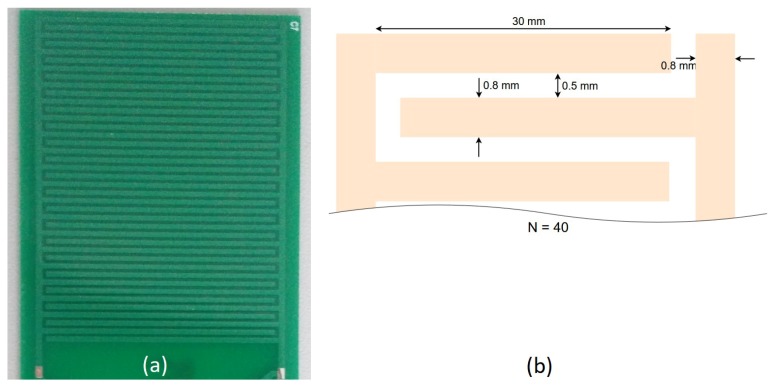
Layout (**a**) and design (**b**) of the interdigitated sensing capacitor.

The measurement solution is based on an interdigitated sensing capacitor printed on the circuit board. The design and layout of the capacitor is shown in Figure 5.

**Figure 6:**
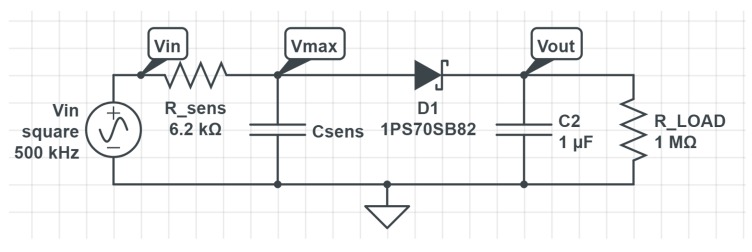
Capacitive moisture sensor.

The thickness of the printed circuit board (PCB) is 0.8 mm and the thickness of the copper tracks on the printed circuit board is 34 μm. The material of the PCB is standard FR4 with $\varepsilon_{r}=4.7$. Sensing capacitor is covered with a 12 μm thick solder mask protection layer. This layer protects copper tracks from corrosion and prevents electrodes’ short circuiting when the sensing capacitor is sunk in soil. The capacitance of the sensing capacitor was measured at 500 kHz using an Agilent E5071C vector network analyzer [17] and was used as a reference measurement for our capacitive moisture sensor. The measured values are 40 pF and 950 pF when the capacitor is in air or submerged in water, respectively. For measuring the capacitance of the sensing capacitor the capacitive moisture sensor, as shown in Figure 6, was developed. The circuit can be divided into two parts: sensing capacitor $C_{\mathrm{sens}}$ and sensor front end, which is made of everything except the sensing capacitor. Together they constitute the capacitive moisture sensor. Diode D1 in the circuit was chosen because it was in stock. Any Schottky barrier diode of reasonable quality and with high enough reverse voltage would be appropriate in this circuit. It should be noted that the models, especially the diode model, were made for this exact diode and the circuit would have to be re-characterized using the same procedure to use another diode. The value of R_sens was chosen experimentally in order to obtain an optimum sensitivity at chosen drive frequencies and given values of $C_{\mathrm{sens}}$. Values of C2 and R_LOAD were also chosen experimentally to be high enough to reduce the output ripple to the desired level, and to obtain a reasonable $V_{\mathrm{out}}$ fall time with a small enough diode drop respectively.

As the microcontroller already contains both a timer and an ADC, the sensor front end only requires two resistors, one diode and one capacitor, and is, thus, extremely compact and cheap. The combined cost of these four components in bulk is around $0.12 –$0.13. We estimate the bulk price of the overall system to be under $10, making the cost of sensor front end negligible. The measurement method works by measuring the amplitude of the voltage on the capacitor via the half wave peak detector. The measured voltage is then divided with the power supply voltage to get results independent of the power supply voltage. The models presented later in the work however assume a 3.3 V supply and will thus have an additional error at a lowered supply voltage. The same models were also made for a supply voltage of 2.8 V and the difference found was insignificant, so the lower supply voltage models were not implemented in the microcontroller firmware.

### 3.1. Capacitive Sensor Model

At the input of the circuit in Figure 6, a 50% duty cycle square wave signal is forced. As the filter is only of the first order, using only the first part of the Fourier series provides an insufficient approximation. For this reason, a simplified analytical model of the circuit was used, ignoring the contribution of loading by diode D1, capacitor C2 and resistor R_LOAD. The charging of the capacitor in the first half period can be expressed as (1).

(1) $$V_{\max}\left(t\right)=V_{\mathrm{DD}}\cdot \left(1-e^{-\frac{t}{R\cdot C}}\right)+V_{\max}\left(0\right)\cdot e^{-\frac{t}{R\cdot C}}.$$

$V_{\max}$ represents voltage on the sensing capacitor and $V_{\mathrm{DD}}$ is the input signal amplitude, which is equal to the supply voltage. *R* is the resistance, *C* is the capacitance and *t* is time. The discharging of the capacitor in the second half period can be expressed as (2).

(2) $$V_{\max}\left(t\right)=V_{\max}\left(0\right)\cdot e^{-\frac{t}{R\cdot C}}.$$

When a steady state is reached on the capacitor, condition (3) holds true and equations (1) and (2) can be merged by inserting equation (2) as V(0) in equation (1), resulting in (4).

(3) $$V_{\max}\left(n\cdot T\right)=V_{\max}\left(\left(n+1\right)\cdot T\right).$$

(4) $$V_{\max}\left(T\right)=V_{\mathrm{DD}}\cdot \left(1-e^{-\frac{D\cdot T}{R\cdot C}}\right)+V_{\max}\left(T\right)\cdot e^{-\frac{D\cdot T}{R\cdot C}}\cdot e^{-\frac{(1-D)\cdot T}{R\cdot C}},$$

where *D* is the duty cycle of the input signal, *T* is signal period and *n* is an arbitrary number. $V_{\max}\left(T\right)$ can be expressed from equation (4) and is presented in (5).

(5) $$\frac{V_{\max}\left(T\right)}{V_{\mathrm{DD}}}=\frac{1-e^{-\frac{D\cdot T}{R\cdot C}}}{1-e^{-\frac{D\cdot T}{R\cdot C}}\cdot e^{-\frac{(1-D)\cdot T}{R\cdot C}}}.$$

$V_{\mathrm{DD}}$ has been moved to the left side of the equation, as the output signal will be divided by its value. As D=0.5 was used, the equation can be further simplified using the rule in (6).

(6) $$\frac{1-z}{1-z^{2}}=\frac{1-z}{\left(1-z)(1+z\right)}=\frac{1}{1+z}.$$

The result of the simplification is:

(7) $$\frac{V_{\max}\left(f\right)}{V_{\mathrm{DD}}}=\frac{1}{1+e^{-\frac{1}{2\cdot f\cdot R\cdot C}}},$$

where *f* is the frequency of the driving signal and was used instead of the period. For *R* the value of R_sens from Figure 6 was used (6.2 kΩ) and for *C* the value of $C_{\mathrm{sens}}$. As the timer used is part of the microcontroller, the measurement sensitivity at different capacitance ranges can be enhanced using different driving frequencies. Frequencies of 500 kHz, 250 kHz and 111.11 kHz were chosen to cover the entire measurement range.

### 3.2. Sensor Front End Characterization

**Figure 7:**
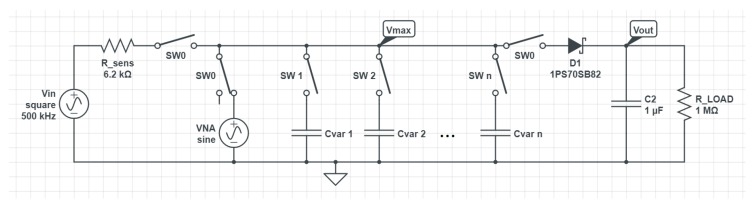
A schematic of the circuit used to characterize the sensor front end.

The sensor front end was characterized using variable capacitors, an Agilent E5071C vector network analyzer [17] and a Keysight DSOX3034T [18] oscilloscope to measure actual $V_{\max}$ on the sensing capacitor and $V_{\mathrm{out}}$ after the peak detector to determine forward voltage on the diode. The diode forward voltage is thus equal to the difference between $V_{\max}$ and $V_{\mathrm{out}}$. To perform the characterization, a special circuit was used. The schematic of this circuit is shown in Figure 7.

Switches in the schematic represent jumpers that were used to connect and disconnect different components. Net names here represent points where oscilloscope probes were connected. The alternating current voltage source named VNA represents the vector network analyzer. All switches named SW0 are toggled at the same time, to get two phases of measurement. Capacitors ${\mathrm{Cvar}}_{1-n}$ are soldered to jumpers and are switched to get the appropriate capacitance. In the first phase of measurement, VNA is connected and appropriate capacitance is set. The oscilloscope probe on Vmax is not disconnected, as it adds to the capacitance, and thus, has to be compensated for. In the second phase VNA is disconnected and sensor front end is connected along with the microcontroller to provide input signal. Maximum voltage is measured with probe on Vmax and a average voltage is measured with probe on Vout. Additionally, voltage on Vout was also measured with ADC on the microcontroller to see if there was any deviation in the values measured. No significant deviation was found.

**Figure 8:**
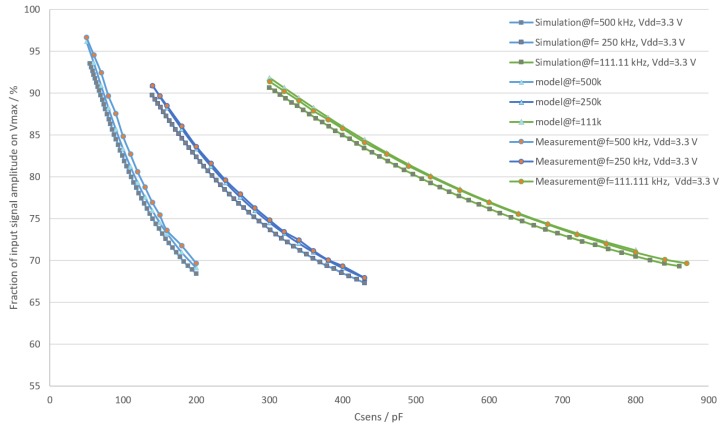
A comparison between results of measurement, simulation and model used.

To ensure the correctness of the sensor front end model and measurements, the circuit was also simulated. For the simulation, Cadence Spectre [19] was used along with the circuit in Figure 6. $V_{\mathrm{out}}$ and $V_{\max}$ were captured as the outputs of the simulation. The comparison between results of simulation, measurement values and the model is displayed in Figure 8.

While measurement values are very close to the model, a correction factor *K* was introduced to eliminate any systematic offset, as shown in (8). Simulation results exhibit a larger offset.

(8) $$\frac{V_{\max}\left(f\right)}{V_{\mathrm{DD}}}=\frac{1}{1+e^{-\frac{K}{2\cdot f\cdot R\cdot C}}}.$$

**Figure 9:**
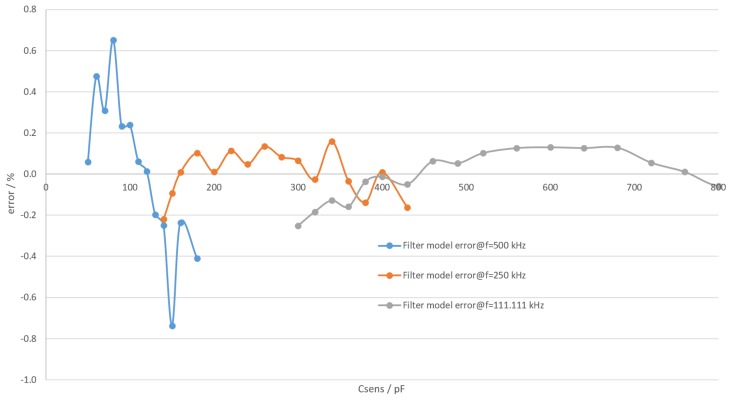
Error of filter model at different frequencies.

The model error is shown in Figure 9. Correction factor values were chosen to minimize integral error. The chosen values were 1.057, 1.011 and 0.99 for 500 kHz, 250 kHz and 111.11 kHz respectively.

The diode forward voltage $V_{\mathrm{diode}}$ was obtained by subtracting peak detector voltage $V_{\mathrm{out}}$ from filter voltage $V_{\max}$ (measurements). The diode forward voltage was modeled using two linear functions with $V_{\mathrm{out}}$ being the input variable for the model. The linear model has the form of $V_{\mathrm{diode}}\left(V_{\mathrm{out}}\right)=A+B\cdot V_{\mathrm{out}}$. To obtain coefficients *A* and *B*, the linear function was fitted to measurement values using the least mean squares method. The input variable of the linear function is measured amplitude on $V_{\mathrm{out}}$, as that is the only known value at the time of measurement. The value given to the model must be in percentage of maximum output amplitude. Calculated values of *A* and *B* are shown in Table 1.

**Figure 10:**
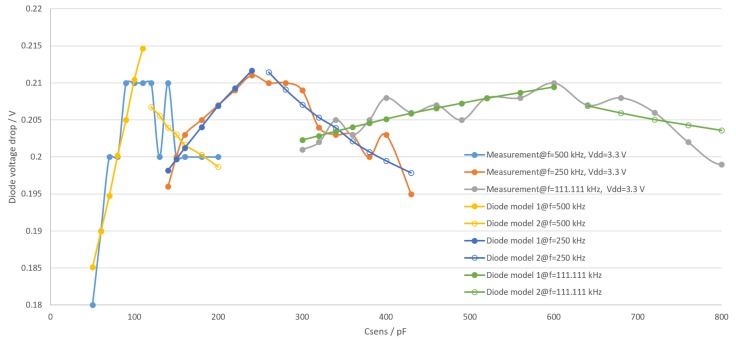
Comparison between measured and modeled diode forward voltage.

Models with number 1 are used at the lower capacitance value of the frequency range or the upper $V_{\mathrm{out}}$ amplitude value. Measured and modeled diode voltage versus the capacitance value is shown in Figure 10.

**Figure 11:**
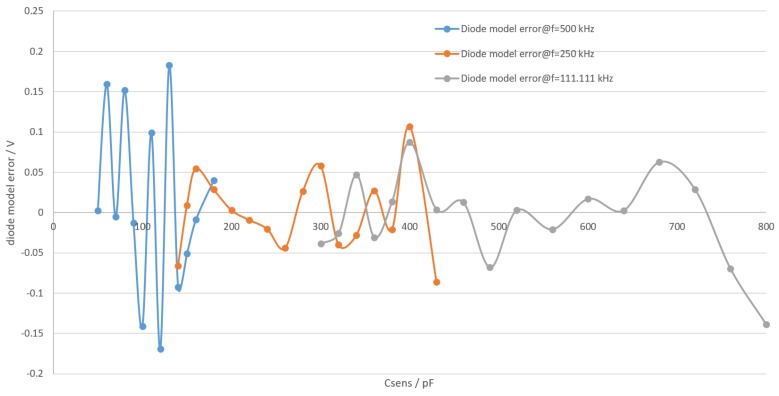
Error of diode forward voltage model.

As there are two models for each frequency, the microcontroller has to choose the appropriate model depending on the measured amplitude and chosen frequency from six available models to calculate the actual voltage on the sensing capacitor. The separation between two models for one frequency is simply done by dividing input variable range in two halves. It should be noted that even though there is a gap between the two models in Figure 10, the two models actually do intersect. Due to the way the models are made, switching between them introduces a discontinuity in the complete model, but this discontinuity is small enough to not be noticeable compared to other model errors in Figure 11. The diode model errors versus capacitance values are shown in Figure 11.

Unfortunately, the diode and filter model errors cannot be canceled out, as the diode forward voltage stays roughly the same with the changing supply voltage, but its contribution in terms of the percentage of filter voltage changes. The changing diode forward voltage is due to changing signal shape on the filter. The signal shape goes from heavily cut square to nearly triangular as capacitance increases. Sensitivity of the sensor front end was also calculated from its characterization results using the general sensor sensitivity formula (9).

(9) $$S=\frac{d\;(\mathrm{sensor}\;\mathrm{output}\;\mathrm{value})}{d\;(\mathrm{sensor}\;\mathrm{input}\;\mathrm{value})}\approx \frac{\Delta \;V_{\mathrm{out}}/V_{\mathrm{DD}}}{\Delta \;C}.$$

**Figure 12:**
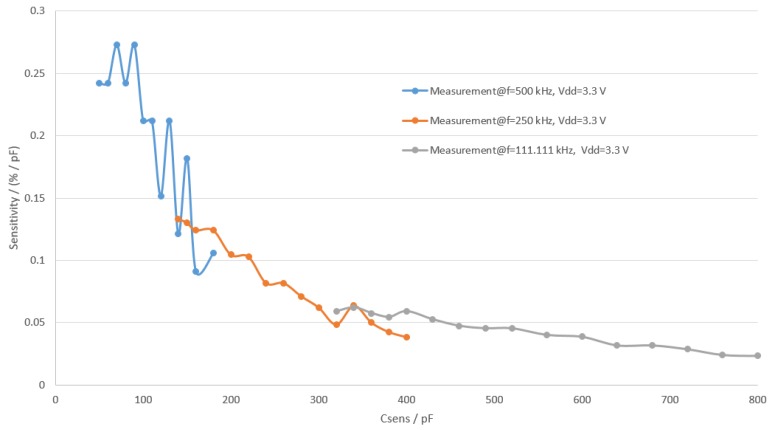
Sensitivity of the sensor front end.

In the formula *S* represents sensitivity. The results of sensitivity calculation are shown in Figure 12. It can be seen from the Figure that frequency transition points were chosen where sensitivity of the higher frequency becomes worse than the sensitivity of the lower frequency. The resolution of the whole measurement system can be calculated from the sensor front end sensitivity by taking into account the resolution of ADC in the microcontroller using (10).

(10) $$Res_{\mathrm{system}}=\frac{Res_{\mathrm{ADC}}}{S}.$$

In the equation, Res represents resolution. The resolution of the ADC was found empirically as the last stable digit when the system was characterized and is 0.02%. This number comes from 12 bits of the ADC combined with 64 times sampling and averaging. The resolution of the system is thus 0.07 pF.

## 4. Measurements and Results Analysis

**Figure 13:**
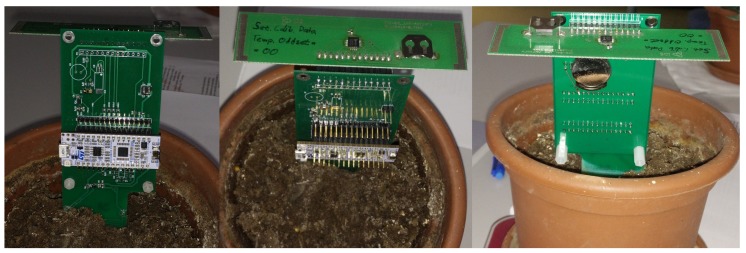
Photos of the measurement system from three different angles.

A flower pot with soil was used to test the device for measuring soil moisture. Photos of the system implanted in a flower pot are shown in Figure 13. It can be noted in the photos that the system was not made water tight, as it was only used indoors. For further tests of the system outdoors it could be coated with epoxy resin to make it waterproof or a 3D printed case could be made to achieve water tightness. A plastic molded case would be appropriate for a mass produced system. Light, relative humidity and temperature were also measured above the flower pot.

### 4.1. Environmental Parameter Measurements

**Figure 14:**
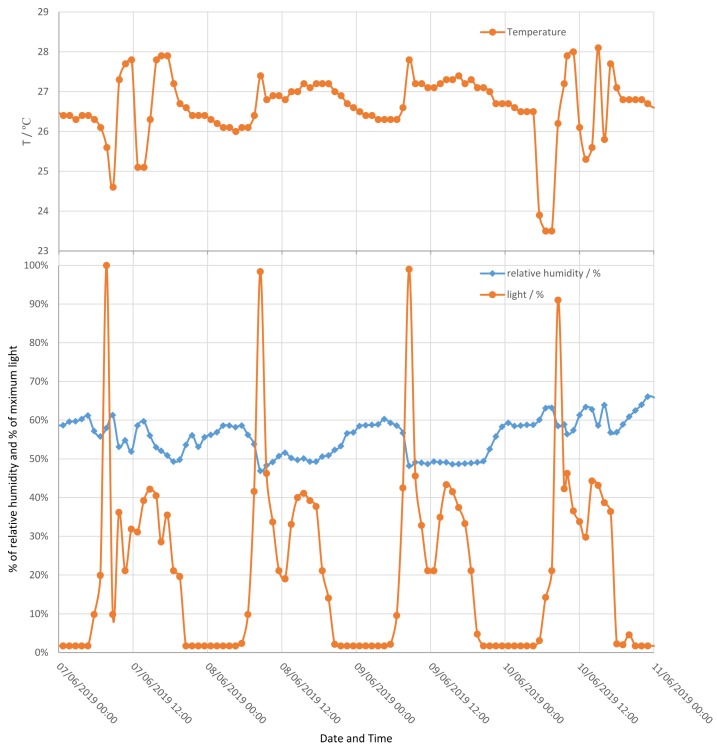
Measurement of relative humidity, temperature and light over four days in the laboratory.

Results of measuring light, temperature and relative humidity can be found in Figure 14. For the light line, the y-axis scale is in terms of percentage of maximum sensor amplitude. The day/night cycle can be seen with direct sunlight in the morning showing as extra high peaks. On the temperature line it can be observed that the temperature in the laboratory was pretty high, with the air conditioner being turned on occasionally.

### 4.2. Capacitance and GWC Measurements

**Figure 15:**
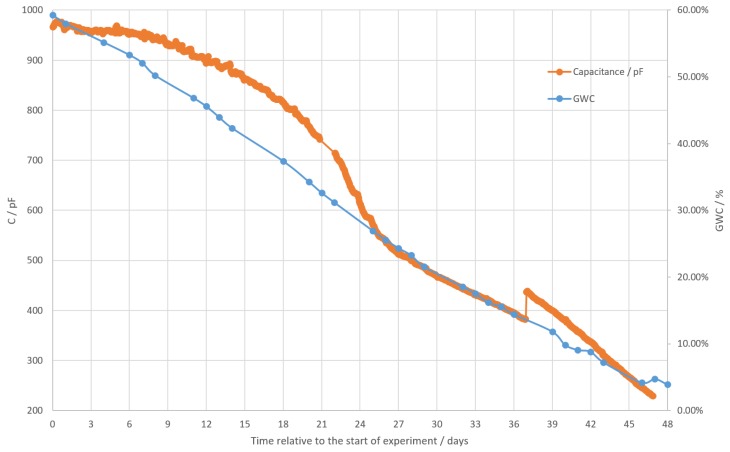
Comparison between capacitive soil moisture measurement and soil gravimetric water content (GWC).

To compare the measured value of capacitance to a reference value, a scale was used to weigh the mass of water in the soil. GWC was then calculated from the mass of water with (11).

(11) $$\mathrm{GWC}=\frac{m_{w}}{m},$$

where $m_{w}$ is the mass of water and *m* is the combined mass of water and dry soil. The comparison between capacitance measurement and GWC is shown in Figure 15.

Soil in the experiment started saturated with water. Its GWC was, thus, at the maximum possible value for this soil. On the graph the soil capacitance starts near the capacitance value of pure water at 960 pF and changes slowly. The rate of water evaporation is fastest at this point, but converting mass to GWC linearizes this. Because of the slow rate of capacitance change, it overshoots the GWC curve at this point. At around 800 pF the capacitance starts decreasing at a faster rate and the two curves converge together again. Near the point of 400 pF there is a discontinuity in the measured capacitance. This discontinuity occurs at the point of switching the measurement range, and thus drive frequency. The possible explanations for it are a frequency dependence of the capacitance of the soil, an issue with model implementation in software or a random event, such as accidental tampering. With additional experiments, this discontinuity should be further investigated in future work. By itself this discontinuity is not an issue, as it could be easily compensated for in capacitance to the GWC model implemented with a lookup table. Capacitance to the GWC model was not developed in the scope of this project, but as can be seen from the graph, GWC could be modeled from capacitance using even a simple linear model using the least squares method, or by using a higher order model, or even a 100 point lookup table. The lookup table model is especially attractive here as it only has to be implemented in software, with no additional hardware cost. The experiment in Figure 15 ends when the soil reaches dry point, and thus covers the entire measurement range. Capacitance to GWC model was not developed here, as it will be unique to the type of soil used and would require having much better reference GWC measurements.

### 4.3. Comparison with Previous Works

The system has been compared to previous works. The comparison of some critical system parameters can be seen in Table 2.

It should be noted that the solution proposed in this work has no systematic error, whereas for example the solution in [5] has over 0.5% of systematic error. Compared to the third solution in the table, our system does have a higher power consumption, but the advantages compared to that system are significant. In addition to soil moisture and temperature measurement, our system also measures relative humidity and light. Our system also has a microcontroller which allows for implementation of advanced models which reduce maximum measurement error compared to [6]. Additionally, the microcontroller allows for addition of new sensors and features. An additional improvement of this system is its semi active nature. The system is able to operate independently without a reader, and thanks to low power consumption, the battery does not have to be replaced for several millions of measurements. If the battery is drained, data are still able to be read from the system.

## 5. Conclusions

In this work a UHF RFID based measurement system for soil moisture, relative humidity, temperature and light was presented. The proposed system could be used in precision agriculture and should, therefore, contribute to future smart village solutions. The measurement system is composed of a well known sensory label SL900A [6] which directly measures temperature, and has an external light sensor attached. Besides these, the study employed BPS230, a commercial low power solution to measure relative humidity, and a novel sensor solution to measure moisture content in soil. The system is based on a capacitive moisture sensor and is composed of only a couple of passive components: some modules already included on the microcontroller and an interdigitated surface capacitor on the PCB. Characteristics of the capacitive measurement system were measured using a network analyzer and an oscilloscope. A model was fitted to the measurement results allowing for an accurate calculation of the capacitance based on the measurement of the amplitude. The proposed model has a maximum error of 0.74% with the average error of just 0.15%. The maximum measurement resolution of the system is 0.07 pF. The measurement system operates independently of the supply voltage and uses different drive signal frequencies to achieve optimum sensitivity throughout the whole measurement range. Due to extremely low power consumption, the presented system can operate with one battery for several years and with the internal capacity for 111 measurement results, the results have to be read out only occasionally.

The capacitive moisture sensor was tested for measuring GWC, and as can be seen in Figure 15, the absolute value of the capacitance drops at the same rate or close to the same rate as GWC. Even a simple linear model will, thus, translate capacitance to GWC with decent accuracy. The discontinuity in the capacitance measurement needs further investigation. Such work, however, is outside of the scope of this paper, as the focus here was to develop the system along with the capacitive moisture sensor and demonstrate the usefulness of the sensor to measure GWC. Therefore, future work should use the sensor in a real environment to better understand the connection between soil capacitance and GWC for different types of soil characteristics (density, porosity, composition, nutrient species, salt content, etc.) and to develop a model to translate capacitance to GWC. A way to compensate for soil parameter variability in the future would be to include other soil parameter measurements, such as impedance, pH and (especially) soil temperature. This could be done with an addition of another temperature sensor to measure temperature of the soil, which would allow compensation of soil capacitance temperature dependence.

It should be noted that if a fine enough lookup table GWC model could be developed, the additional error after the capacitance error would be inconsequential. Thus, errors in Figure 9 and Figure 11 would be the only errors of the measurement. Resolution of GWC measurement will depend on the soil characteristics, as they affect the sensitivity of capacitance relative to GWC. Using the sensitivity of soil capacitance to GWC for soil used in this work, the resolution can be estimated to 0.01%, with sensitivity being 20 pF/% and resolution at that point being 0.21 pF. Furthermore using data from humidity, temperature and light sensors algorithms should be developed to find optimum times to fertilize, spray pesticides and other chemicals and water the crops. Data from these sensors combined with a visual inspection should be sufficient to know the exact growth state of the crops. In cases such as smart village and smart agriculture applications, the data produced by these sensors would be stored for future reference and analyzed to find valuable information for successful farming. For that, very different technologies could be implemented, such as machine-learning algorithms. But such things are a subject for future work.

## Figures and Tables

**Table 1:** Diode model coefficient.

Frequency	Model Number	*A*	*B*
500 kHz	1	0.366	−0.00199
500 kHz	2	0.150	0.00076
250 kHz	1	0.295	−0.00114
250 kHz	2	0.109	0.00142
111.11 kHz	1	0.243	−0.00046
111.11 kHz	2	0.153	0.00071

**Table 2:** Comparison between this work and previous works.

Parameter	This Work	[5]	[6] 2nd Solution
Carrier frequency	860–960 MHz	13.56 MHz	860–960 MHz
Protocol	EPC Gen2	ISO 15693	EPC Gen2
Power consumption	340 µA @ 3.3 V	1240 µA @ 3.3 V	180 µA @ 3 V
Max. error ΔC/C(%)	<0.8% @ 50–950 pF	<2% @ 50–270 pF	<5.15 % @ 15–88 pF
Active / Passive	semi active	passive	passive
